# Disease to Dirt: The Biology of Microbial Amyloids

**DOI:** 10.1371/journal.ppat.1003740

**Published:** 2013-11-21

**Authors:** David A. Hufnagel, Çagla Tükel, Matthew R. Chapman

**Affiliations:** 1 Department of Molecular, Cellular, and Developmental Biology, University of Michigan, Ann Arbor, Michigan, United States of America; 2 Department of Microbiology and Immunology, School of Medicine, Temple University, Philadelphia, Pennsylvania, United States of America; Washington University School of Medicine, United States of America

Amyloid fibers are β-sheet-rich protein polymers that are highly resistant to denaturation. The distinguishing amyloid fold can be adopted by a variety of proteins, without a shared primary structure, and is found in nearly all cell types. Despite the fact that amyloids have a richly informed scientific history, the diverse biology contributed by amyloids is only beginning to be appreciated. Initial amyloid studies focused on the intimate association of amyloid formation with cytotoxicity and neurodegenerative diseases like Alzheimer's, Huntington's, and the prion encephalopathies. Despite amyloid's somewhat sinister past, recent work on “functional” amyloids has revealed numerous ways that amyloids contribute to normal cellular biology [1]. Included among the activities in which amyloids participate are melanin production, the ability to act as non-Mendelian inheritable genetic elements, and as extracellular molecular scaffolds that hold bacterial communities together. The amyloid fold is tailor-made for the extracellular space, as amyloid polymers can self-assemble without requiring exogenous energy and the polymers are resistant to a slew of harsh denaturants that would devastate most protein folds. This article will address questions involving the various roles of bacterial amyloids in host, polymicrobial, and environmental interactions.

## Are Bacterially Produced Amyloids Widespread or an Anomalous Extracellular Structure?

Many bacterial species produce extracellular amyloid fibers, where they typically function as part of a complex protein and polysaccharide matrix that protects communities of cells. *Escherichia coli, Salmonella enterica* serovar Typhimurium, *Bacillus subtilis*, *Staphylococcus aureus*, and *Mycobacterium tuberculosis*, among many others, produce extracellular amyloid fibers [2]–[5]. Between 5–40% of species isolated from natural biofilms (seawater, sludge, and drinking water) produce amyloids, demonstrating the widespread occurrence of this extracellular structure [6]. Amyloid production in biofilms provides structure for floating biofilms in *B. subtilis* and *E. coli* and for shear and matrix-degrading enzyme-resistant biofilms in *S. aureus* [3], [4], [7]. The functions and presence of bacterial amyloids were first unraveled in the study of curli fibers made by *E. coli* [2].

Curli are a functional bacterial amyloid expressed by a variety of enteric bacteria and utilized as a community resource in biofilms. The curli biogenesis machinery is encoded within two *csg* (curli specific gene) operons that include a master transcriptional regulator of biofilm extracellular matrix (ECM) components called CsgD. Encoded in the same operon as CsgD are two chaperone-like proteins (CsgE and CsgF) that coordinate with CsgG to form a unique secretion system that transports curli subunits to the cell surface. At the cell surface, the major curli subunit called CsgA is anchored to the cell and nucleated into a β-rich amyloid polymer by the CsgB protein [8]. The curli assembly machinery is widespread, as *csg* homologs are found within four phylum, Bacteroidetes, Proteobacter, Firmicutes, and Thermodesulfobacteria, and nine different classes of sequenced bacteria [9]. CsgA's imperfect glutamine and asparagine-rich repeating units, which are necessary for amyloid polymerization, are conserved in these differing versions of CsgA. Additionally, there is striking continuity among *csg* ORFs in disparate bacterial species, implying that the identified *csg* homologs are not pseudogenes. The *csgDEFG/csgBAC* intergenic region is highly conserved in different *E. coli* isolates, having a 98.6% average pair-wise identity in 284 different *E. coli* strains [10]. Along with *asnS/ompF* and *uspC/flhDC*, the *csgDEFG/csgBAC* intergenic sequence can be used to construct a phylogenetic tree with a more accurate prediction of *E. coli* phylogeny with fewer base pairs than the traditional MLST method [10]. The expansive nature of *csg* genes and bacterial amyloids discovered in a myriad of species points to amyloids being a common and important part of the communal bacterial lifestyle.

## What Role Do Curli Play in Host-Bacteria Interactions?

There is a complicated interplay between the host immune system and curli fibers. Curli are often maximally expressed in the laboratory when bacteria are grown under low temperature and low osmolarity, conditions that are not readily associated with host environments. However, both *S.* Typhimurium and *E. coli* express curli *in vivo* during infection, and the regulation of curli expression is incredibly complex [11], [12].

**Figure 1:**
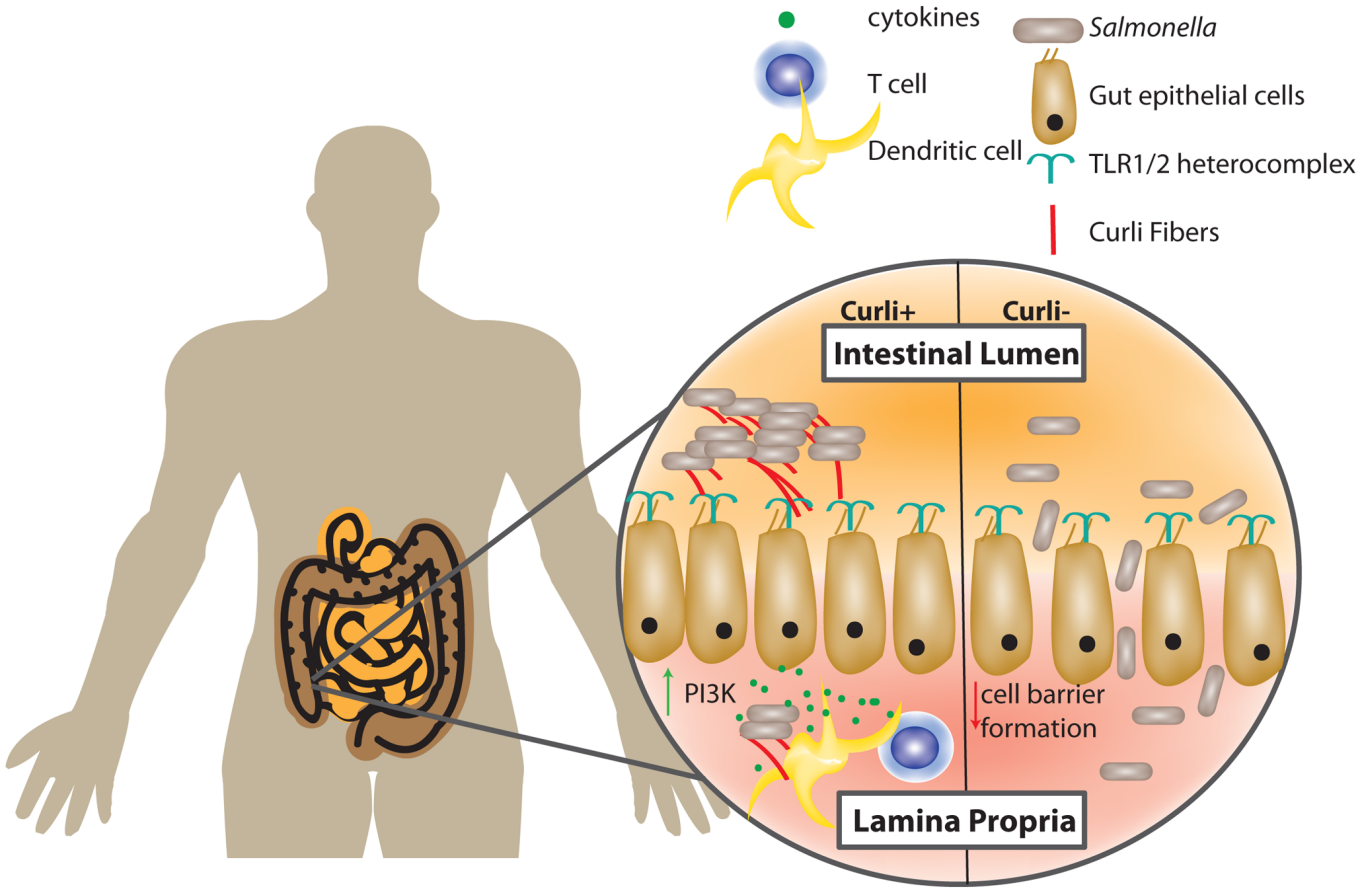
Gut epithelial cells and the host immune system recognize curli fibers. *Salmonella* and *E. coli* are intestinal-dwelling bacteria whose curli fibers are recognized by host epithelial cells. The TLR1/2 heterocomplex recognizes the mature curli fiber and causes a signaling cascade in host cells. Recognition of curli results in an increase in PI3K in gut epithelial cells and increases gut epithelial cell barrier formation. Curliated *Salmonella* elicit an increase in cytokine production by T cells and dendritic cells. *Salmonella* curli mutants cause decreased epithelial cell barrier formation and lead to increased extraintestinal titers of *Salmonella*.

Curli amyloid fibers are recognized by the host immune system, culminating in a signaling cascade that alters the interaction between bacteria and host. Various immune cells recognize curli fibers via the TLR2/TLR1/CD14 heterocomplex [13]. Intestinal epithelial cells directly respond to curli fibers on *S.* Typhimurium, leading to an increase in PI3K expression and a subsequent decrease in epithelial cell barrier permeability. Conversely, infection with non-curliated bacteria results in an epithelial barrier with increased permeability. The more permeable epithelial barrier allows for higher bacterial titers in the cecal tissue and mesenteric lymph nodes (Figure 1) [14], [15]. However, it is also important to note that although the barrier is reinforced, the curliated pathogens can overcome the protective immune response and cause inflammation. Interestingly, when curliated cells cross the epithelial barrier, multiple immune cells such as macrophages, dendritic cells, and T cells respond by upregulating various proinflammatory cytokines including IL-6, IL-23, IL-17A, and IL-22 [16]. Collectively, this work suggests that commensal curliated bacteria may exert protective effects on the epithelial barrier via TLR2 activation, but additional work is needed to elucidate the complex interplay of curli, enteric bacteria, gut epithelial cells, and immune cells.

It is unknown whether curli interact with other pathogenic amyloids in the host environment. Intravenous injection of curli (along with other amyloids) into mice increases the occurrence of amyloid protein A amyloidosis in spleens isolated from mice [17]. Some interesting studies have looked at curli interaction with other amyloids *in vitro*, as islet amyloid polypeptide cannot decrease the lag time of CsgA amyloid formation, but CsgA or CsgB addition to PAP_248-286_ decreased lag time and increased the elongation rate of amyloid formation [18], [19]. Clearly, more work needs to be done to fully understand the relationship between functional amyloids like curli and those produced by humans that are associated with protein misfolding and disease.

Curli also play a role in the development of urinary tract infections by *E. coli*. Curliated uropathogenic *E. coli* (UPEC) have increased survival during co-incubation with bladder epithelial cells compared to non-curliated variants, and this interaction appears to be due to curli interacting with the human antimicrobial peptide LL-37 [20]. LL-37 normally perturbs membranes causing lysis, but the presence of polymerized curli fibers leads to inhibition of the antimicrobial activity. LL-37 can alternatively inhibit amyloid polymerization of CsgA *in vitro*, which provides evidence that LL-37 may be caught in binding to the curli fibers during their polymerization process vs. accessing the bacterial cell membrane to cause lysis in a curli-null strain. Curli also participate in bladder colonization, as deletion of curli genes or small-molecule inhibitors of curli polymerization modestly decreases *E. coli* colonization of mouse bladders at six hours post-infection [7]. The specific and complicated interaction of curli and the immune system demonstrates the importance of functional amyloids in host-bacteria interactions.

## How Are Curli Utilized by Bacteria outside of the Host?

Curli are a community resource that can increase fitness in an array of environmental conditions encountered during the bacterial life cycle [8]. As enterics, *Salmonella* spp. and *E. coli* must be equipped to persist in multiple environments both inside and outside the host, including the digestive tracts of animals. Life in the intestines can lead to passage through the host and emergence to many different conditions, including conditions with decreased nutrient concentration and temperature.

**Figure 2:**
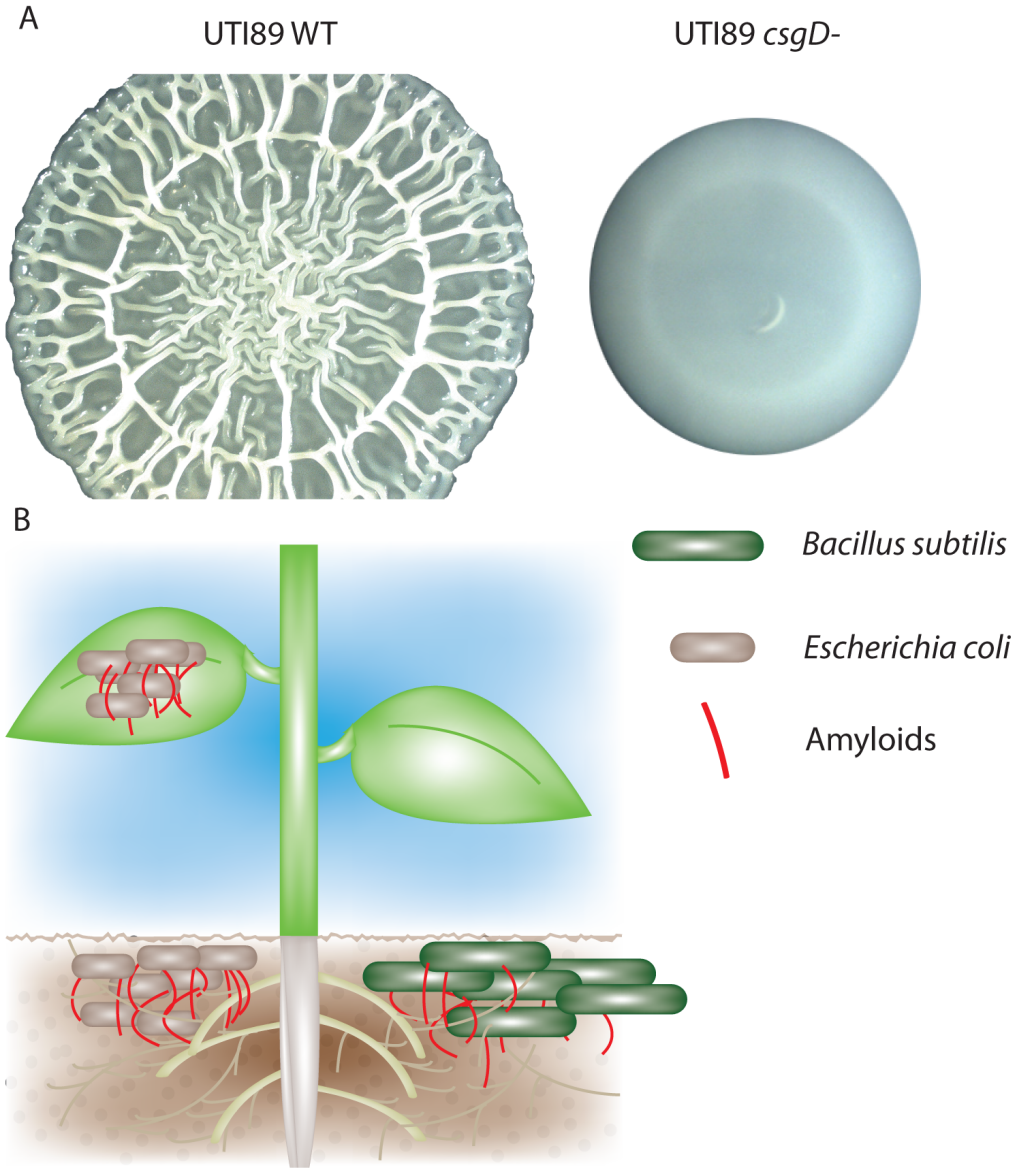
Bacterial amyloids are utilized in multiple environments. A) *E. coli* UPEC (UTI89) rugose biofilms form a complex spreading and wrinkling pattern that is dependent on the biofilm regulator CsgD. *csgD* mutant colonies do not spread or wrinkle. B) Curli are utilized by *E. coli* for increased adherence to lettuce roots and spinach leaves. *Bacillus subtilis* amyloid fiber proteins and biofilm formation are induced in the presence of tomato plant root exudate.

*Salmonella* and certain species of *E. coli* form ornate wrinkled or rugose colony biofilms (also known as rdar [red, dry, and rough]) on low-salt agar plates at low temperatures. Rugose biofilms are dependent on the extracellular structures, cellulose and curli, that are controlled by the global regulator of biofilm, CsgD (Figure 2A) [21]. Rugose biofilms of UPEC have two distinct cell populations: a top layer at the colony-air interface containing curliated cells, and a non-curliated interior population. The development of these two populations is triggered by exposure to reactive oxygen stress, either through superoxide accumulation or through the addition of iron in *E. coli*, *Salmonella*, and *Citrobacter* [22]. Rugose biofilms provide resistance to desiccation, as WT *Salmonella* rugose colonies incubated at room temperature for three and nine months had greatly increased survival compared to curli and cellulose mutants [23].

Polymicrobial communities can be readily found in the soil or associated directly with plant roots. Amyloids play important roles in bacterial association with plant roots and leaves (Figure 2B). Curli increase the adherence of pathogenic *E. coli* O157:H7 to spinach leaves [24]. Hou *et al.* found that *E. coli csgA* mutants have decreased attachment to lettuce roots compared to WT *E. coli* in a hydroponic assay system used to replicate the interactions between bacteria and the rhizosphere (the area of soil directly surrounding plant roots that is rich in interspecies signaling and plant exudate) [25]. Microbial amyloids appear to have broad interaction with the rhizosphere: *B. subtilis* pellicle biofilm formation and the *tapA* gene (which encodes a protein important for bacterial amyloid formation) are upregulated by tomato plant root exudate [26].

Because polymicrobial interactions are commonplace, it is rare for natural environmental bacterial communities to be composed of a single bacterial species. Polymicrobial community development can be driven by interactions between extracellular fibers like curli. *Salmonella*, *Citrobacter*, *Shewanella*, and *E. coli* CsgA fibers can interact *in vitro* [27]. These species also have interactions between the curli templator, CsgB, and the main structural subunit of curli, CsgA. *Salmonella csgA* and *E. coli csgB* mutants, along with *Salmonella csgB* and *E. coli csgA* mutants, can complement each other leading to curli fiber formation, pellicle biofilm formation, and increased surface attachment [27]. Interactions with plant leaves, roots, and other bacterial species lends support to the notion that curli are a community resource that can be deployed for multiple functions in the environment. Bacterial amyloids influence the interaction between host and microbe in many different ways, including augmentation of resistance to host defense and increasing the attachment of microbes to each other and to common food sources of humans.

Microbial amyloids are a ubiquitous community resource utilized by bacteria both inside and outside of the host. Amyloid fibers have functions ranging from tightening gut epithelial cell junctions to increasing community and surface adherence. The role of curli in increasing adherence to various surfaces and resisting various stresses in the environment may shed light on how curli impact the development of communities within the host. Curli may increase *E. coli's* ability to associate with one another or host tissues in the gastrointestinal tract or during pathogenesis. The complex interplay between microbial amyloids and the host is just beginning to be unraveled. Continued elucidation of bacterial amyloid biology will further our knowledge of the role of functional amyloids in disease, commensal-host interaction, and microbial community development. This knowledge could lead to potential therapies or probiotics to beneficially exploit amyloid formation in the host and environment.
